# The Importance of Hazard Analysis by Critical Control Point for Effective Pathogen Control in Animal Feed: Assessment of *Salmonella* Control in Feed Production in Sweden, 1982–2005

**DOI:** 10.1089/fpd.2023.0067

**Published:** 2023-11-29

**Authors:** Martin Wierup

**Affiliations:** Department of Biomedical Science and Veterinary Public Health (BVF), Swedish University of Agricultural Sciences (SLU), Uppsala, Sweden.

**Keywords:** HACCP, feed mill, *Salmonella*, animal feed, soy meal, poultry, swine

## Abstract

This study is the first to show that Hazard Analysis by Critical Control Point (HACCP)-based monitoring can be an effective tool for ensuring *Salmonella*-safe feed, by virtually eliminating feedborne *Salmonella* infection even in broiler production. Data from the control of *Salmonella* in feed and food animal production during 1982–2005, showed that conventional endpoint testing in feed mills did not ensure a *Salmonella*-safe feed, and in one feed mill failed to detect *Salmonella* contamination, resulting in the feed infecting 80 out of 197 (40.6%) recipient broiler flocks. Following implementation in 1991 of a HACCP-based control in feed mills, the annual number of samples tested at specified critical control points during a 15-year period increased from ∼4400 to 10,000, while the proportion of *Salmonella*-contaminated samples decreased from 2.0% to 0.3%. Thus, introduction of HACCP was followed by a dramatic decrease, from 40 to <5, in the annual number of *Salmonella*-infected broiler flocks identified by preslaughter monitoring. Incidence has generally remained at that low level, despite production since 1980 increasing from 39 to 112 million chickens per year. Feed mills start using soymeal with an unsafe *Salmonella* status and possibly with a suboptimal HACCP control, increased their level of *Salmonella*-contaminated HACCP samples, and their feed subsequently infected 78 swine-producing herds. The results also show that the HACCP concept can be an effective tool to supply feed mills with *Salmonella*-safe feed ingredients as demonstrated for a soybean crushing plant, which produced *Salmonella*-safe soymeal over a 19-year period despite frequent (34%) and highly varied (92 different serovars) *Salmonella* contamination in samples from incoming soybean. Similar results are reported for a plant producing rapeseed meal. It is emphasized that the achievements described through use of the HACCP required interventions of relevant preventive biosecurity measures and corrective actions when the HACCP-based monitoring identified *Salmonella* contamination.

## Introduction

The Hazard Analysis by Critical Control Point (HACCP) concept was first developed in the 1960s by the U.S. National Aeronautics and Space Administration, to ensure the safety of foods for space flights (Lachance, 1997). HACCP was later recognized as an effective alternative to conventional *endpoint-testing* and recommended as a food safety management tool by various international organizations such as World Health Organization (WHO) and Food and Agriculture Organization of the United Nations (FAO) (Bryan and World Health Organization, 1992; WHO, 1999). Use of HACCP is currently prescribed in national food safety legislation in many countries and is also required by private companies (Ropkins and Beck, 2000; Weinroth et al., 2018).

In the European Union (EU), requirements for HACCP-based food safety procedures have been in place since 2004 (EC, 2004) and corresponding requirements were introduced for animal feed production in the following year (EC, 2005). The need to improve the safety of animal feed, the first link in the animal-derived food chain, was revealed by serious food safety crises caused in particular by bovine spongiform encephalopathy (EFSA, 2022a) and the pandemic spread of *Salmonella* Enteritidis from poultry production (Rodrigue et al., 1990).

Previous evaluations of the impact of HACCP monitoring on food safety have focused on the implementation of HACCP, in particular on the procedures and time requirements for training staff (Anandappa, 2013; Ehiri, 2018; Ropkins and Beck, 2000). Introduction of HACCP in the United States is estimated to have reduced foodborne illnesses by 20% in the 7 years after its implementation (Weinroth et al., 2018), but no dedicated study has examined the impact of HACCP on food or feed safety. One explanation for the lack of such studies, for example, on HACCP control of *Salmonella*, is the analytical challenge caused by endemic occurrence and presence of several serovars of *Salmonella* simultaneously in different parts of the food chain, as is currently the case for example, in several EU Member States (EFSA, 2022b). However, it is not the case in Sweden, because active control of *Salmonella* in feed and animal production has been in place since the 1960s (EFSA, 2022a; SVA, 2022).

Historical data obtained from *Salmonella* monitoring in Swedish feed and animal production were analyzed in this study to obtain scientific evidence on whether HACCP-based monitoring with associated interventions is an effective tool for reducing *Salmonella* contamination in the feed mill production of the finished feed and, indirectly, feedborne *Salmonella* infections in broiler and swine production.

## Materials and Methods

### General

The data analyzed comprised documented experiences and outbreak events from preslaughter monitoring and control of *Salmonella* in Sweden during the period 1982–2005. By the end of that period, virtually *Salmonella*-free animal and feed production had been achieved and maintained by applying a long-term statutory zero-acceptance policy for any serovar of *Salmonella* in feed, production animals, and food (Wierup et al., 2021). Key elements in *Salmonella* monitoring in commercial feed production included downstream checks for *Salmonella* infections in herds receiving feed found to be *Salmonella*-contaminated and upstream checks for *Salmonella* contamination of feed mills delivering feed to animal herds found to be *Salmonella*-infected.

A first analysis examined the ability of endpoint testing in feed mills, that is, non-HACCP monitoring, to ensure detection of *Salmonella* contamination in finished feed and thus prevent delivery of such feed to animal producers. A second analysis examined changes in the level of *Salmonella* contamination within feed mills following the introduction of HACCP, while a third analysis examined subsequent changes in the incidence of *Salmonella*-infected broiler flocks. A fourth and final analysis assessed the importance of effective HACCP control of a high-risk feed material (soymeal) in avoiding *Salmonella* infection in pig production.

## Results

### Analysis 1. Ability of non-HACCP monitoring in feed mills to ensure *Salmonella*-free broiler feed.

#### *Salmonella* monitoring in feed mills

Before the introduction of HACCP-based monitoring in the Swedish feed mills in 1991, monitoring for *Salmonella* contamination had been based solely on bacteriological testing of certain risk feed ingredients like soymeal since the 1970s and, since 1987, on endpoint testing of nonheat treated finished feed (Cerenius, 2010).

#### *Salmonella* monitoring in Swedish broiler production

Due to problems with human outbreaks of salmonellosis originating from *Salmonella*-infected broilers, in 1970, the Swedish broiler industry introduced voluntary monitoring of *Salmonella* in broiler flocks preslaughter. During the period July 1, 1981, to December 31, 1984, 1% of each participating broiler flock was tested on two occasions, 1–2 weeks after hatch, and 1–2 weeks before slaughter. From January 1985, testing was performed only once, with 15% of dead and culled birds tested 1–2 weeks before slaughter. Flocks found to be *Salmonella*-infected were destroyed, with 90% of the costs paid by the state. Approximately 10% of broiler producers did not join the voluntary scheme, but they were forced to join a revised scheme from 1984 onward, when the state made preslaughter testing of broiler flocks mandatory and also withdrew its economic compensation, which was replaced by producer-funded insurance (SVA, 2022; Wierup et al., 1995).

#### Seven-month feedborne outbreak of *Salmonella*

During roughly a 7-month period, from December 1981 to June 1982, *Salmonella* Livingstone was spread by feed from one feed mill to broiler-producing farms in southern Sweden and detected in statutory monitoring of the broiler flocks (Wierup et al., 1988). The outbreak resulted in an intensive search for *Salmonella* contamination in the supplying feed mill.

Despite increased monitoring for *Salmonella* in the feed mill, in particular of finished feed after the heat treatment, no *Salmonella* contamination was identified until *Salmonella* Livingstone was detected within the production line at the end of the 7-month period (Cerenius, 2010). Although negative results for *Salmonella* contamination were obtained in testing at the feed mill, 80 out of 197 broiler flocks supplied with feed (40.6%) were infected by *Salmonella* Livingstone during the outbreak period. All these flocks received feed only from that feed mill and all chicks were supplied by the same hatchery, which was under continuous monitoring for *Salmonella*, with negative results (Wierup et al., 1992). The rate of infected flocks would probably have been even higher in the absence of competitive exclusion, which was successfully introduced for flocks from 15 selected producers to prevent further outbreaks of *Salmonella* (Wierup et al., 1988).

### Analysis 2. Introduction of HACCP-based monitoring in feed mills

Because of the feedborne outbreak described above, the feed industry, through its foundation “Veterinary Feed Control,” decided to introduce HACCP-based self-monitoring in 1991. Three years later, the state made such monitoring mandatory (Cerenius, 2010), and in 2005, it became a statutory requirement in the EU (EC, 2005). In Sweden, the industry continually updates the monitoring requirements in its National Guidelines (Foder och Spannmål, 2022). For broiler feed, the legal requirements include heat treatment and weekly sampling at the following five critical control points: material from intake pit/bottom part of elevator (1), dust from aspiration system/filter (2), dust from room for pellet coolers (3), dust from top of all pellet coolers (4), and dust from top of bin for finished feed (5). The samples taken at those mandatory control points are analyzed according to standard procedures at the National Veterinary Institute (SVA). Additional sampling may be performed as part of the HACCP plan for each feed factory.

**FIG. 1.**
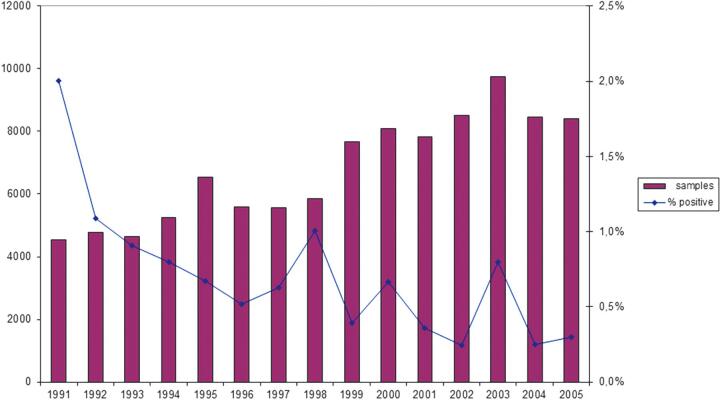
(Bars) Total number of samples from environmental monitoring in all approved Swedish feed mills in the 15-year period following introduction of HACCP-based monitoring for *Salmonella* contamination in 1991 and (dots) percentage of samples found to be contaminated with *Salmonella*. (Source: National Veterinary Institute (SVA), printed by permission from M. Thelander. HACCP, Hazard Analysis by Critical Control Point.

The number of samples analyzed for all nationally approved commercial feed mills in Sweden (15–18 with an annual production volume ranging from 50- to >200 thousand tons) and the proportion of samples found to be *Salmonella*-contaminated during the 15 years after introduction of HACCP-based monitoring are presented (Fig. 1). As can be seen, the annual number of samples analyzed gradually increased from ∼4400 to 10,000 over the period, while the proportion of *Salmonella*-positive samples decreased from ∼2.0% to 0.3%.

### Analysis 3. Annual incidence of *Salmonella*-infected broiler flocks following the introduction of mandatory HACCP-based monitoring of *Salmonella* in feed production, mandatory preslaughter monitoring of broilers for *Salmonella*, and withdrawal of state compensation for destruction of *Salmonella*-infected flocks

**FIG. 2.**
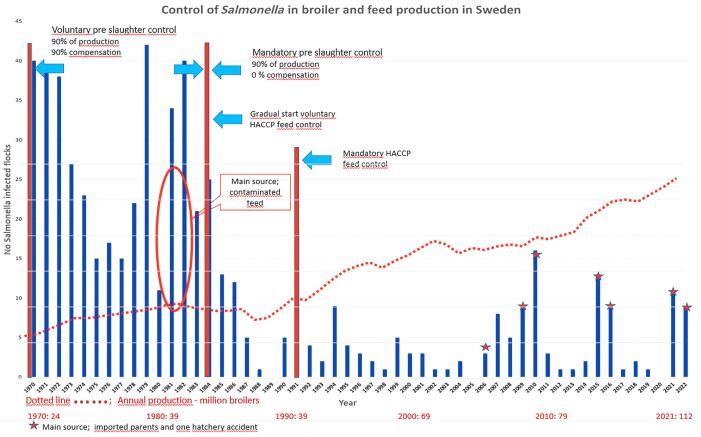
Number of Swedish broiler flocks found to be *Salmonella*-infected in relation to introduction of voluntary (1970) and mandatory (1984) preslaughter monitoring, volume of broiler production, introduction of voluntary (1984) and mandatory (1991) HACCP-based feed monitoring for *Salmonella*, and some major sources of infection.

Following cost increases for national compensation for voluntary monitoring of *Salmonella* in broiler production, in particular due to the feedborne outbreak described above, the compensation scheme (covering 90% of producers' costs) was withdrawn and voluntary control of broiler flocks for *Salmonella* (covering 90% of production) became mandatory in 1984 (Fig. 2).

The annual number of broiler flocks found to be *Salmonella*-infected in preslaughter monitoring as well as significant control measures introduced between 1970 and 2022 are presented (Fig. 2). In addition poultry feed was heat-treated since 1972 (Cerenius, 2010). Initially, there was no particular change in the annual incidence during the 4 years after withdrawal of state compensation and statutory monitoring of all producers. However, a considerable decrease in the annual incidence began to emerge after the introduction of HACCP-based *Salmonella* monitoring in feed mills. This decrease started in 1988, when HACCP-based monitoring began to be gradually introduced, and eventually reached a level of <5 to 10 infected flocks per year. Even though the volume of broiler production in Sweden has increased by 310% over recent decades, from ∼39 million in 1990 to 112 million in 2021 (Jordbruksverket, 2021; Lannhard-Öberg, 2018), low numerical incidence of *Salmonella* has largely been maintained up to the present day, apart from some temporary infection events at hatcheries (SVA, 2022).

### Analysis 4. Impact of effective HACCP monitoring of high-risk feed material (soymeal) to avoid *Salmonella* infection in pig production

This assessment covered a 2-year period (2004–2005) in which *Salmonella* was spread via feed from two feed mills to 49 and 29 swine-producing herds, respectively. Both feed mills belonged to the same company (Company A). A major part of the assessment was based on results from statutory surveillance, before use in feed mills, of vegetable proteins identified as high-risk feed ingredients for *Salmonella* contamination and from HACCP monitoring of Swedish feed mills. The outbreak was analyzed in a national inquiry (Wierup, 2006), and major results were published separately (Wierup and Häggblom, 2010).

**FIG. 3.**
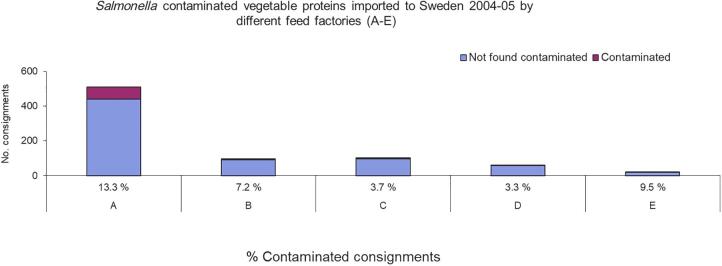
Incidence of *Salmonella*-contaminated consignments of imported vegetable proteins detected at testing before use in feed production at feed mills owned by different companies in Sweden (Companies A–E). Modified from Wierup (2006).

Before the outbreaks, Company A had largely replaced a *Salmonella*-safe source of soymeal with imports from soybean crushing plants with a history of unknown or frequent *Salmonella* contamination. The results from monitoring *Salmonella* contamination in these imports and other imported vegetable proteins were compared (using χ^2^ tests) to the monitoring results from the feed mills of the other feed-producing companies (Companies B–E) registered in Sweden (Fig. 3). The relative risk of *Salmonella*-contaminated consignments of vegetable proteins was found to be 2.4 times higher (*p* < 0.0006) for Company A mills than for the mills of Companies B–E, which all mainly imported soymeal from a crushing plant with a documented low risk of *Salmonella* contamination (Wierup and Kristoffersen, 2014). Company A mills was responsible for 54% of all consignments of vegetable proteins imported to Sweden, which contained 85% of the total number of *Salmonella*-contaminated samples and 71% of the different *Salmonella* serovars isolated.

**FIG. 4.**
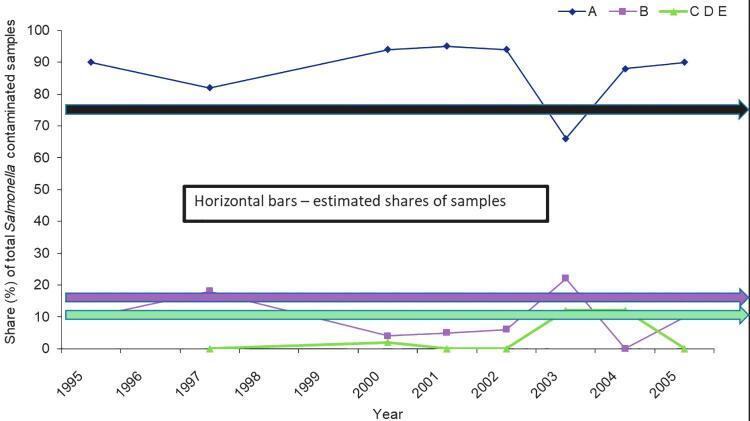
Annual proportion of *Salmonella*-contaminated samples identified in HACCP monitoring in Sweden at feed mills belonging to different companies (Companies A–E) in relation to their share of samples tested. Modified from Wierup (2006).

The proportion of *Salmonella*-contaminated samples identified in mandatory HACCP-based monitoring within the feed mills, as a proportion of their share of samples (i.e., volume of feed produced), was significantly higher for Company A mills than for mills owned by the other companies (Fig. 4). Company A mills produced on average 75% of the national feed volume and provided 87% (exact data not shown) of the contaminated samples. For the other mills, the proportion of *Salmonella*-contaminated samples was lower than their share of production (10% compared with 15% for Company B mills, and 3% compared with 10% for Company C–E mills (Fig. 4).

## Discussion

This study is the first to show that HACCP-based monitoring can be an effective tool for ensuring *Salmonella*-safe feed, thereby minimizing or even eliminating feed as a major source of *Salmonella* in the animal-derived food chain (EFSA, 2008). The analysis was based on long-term data originating from active monitoring and control of *Salmonella* in Sweden, which has resulted in a virtually *Salmonella*-free animal production. Because commercial feed production is technically similar in most countries and is also similar in terms of the feed ingredients used and the risk of *Salmonella* contamination (EFSA, 2008), the experiences from Sweden are likely also applicable to other countries.

The analysis revealed inability of endpoint testing of the finished feed to ensure freedom from *Salmonella* contamination. Despite negative results for *Salmonella* contamination during frequent testing of finished feed, during a 7-month period, a feed mill delivered feed to 197 broiler flocks, of which 80 (40.6%) were found to be infected by *Salmonella* Livingstone. This serovar was eventually isolated from production lines at the feed mill after other sources of infection, including from breeding flocks, had been ruled out (Cerenius, 2010; Wierup et al., 1995).

These results demonstrate that *Salmonella* monitoring in feed production is necessary to detect and ensure freedom from any concentration of the pathogen, because even very low numbers of *Salmonella* bacteria in the feed can result in young chicks readily becoming infected following per-oral exposure. This risk was early observed by Nurmi and Rantala (1973) and for their development of the competitive exclusion concept, successfully applied also in Sweden as referred above. The results also show that in practice, effective *Salmonella* control cannot be achieved simply by testing large volumes of the finished feed.

The introduction of HACCP-based monitoring of *Salmonella* at a specified minimum number of control points increased the number of samples tested for *Salmonella* contamination (Fig. 1). Data on the proportion samples found to be *Salmonella*-contaminated before the introduction of HACCP are not available, but the increased sampling logically detected previously unidentified spots of contamination, which increased that proportion. However, the proportion of *Salmonella*-positive samples dramatically decreased following the introduction of HACCP, from ∼2.0% to 0.3% (Fig. 1), likely leading to a corresponding reduction in the risk of *Salmonella* contamination in the finished feed. Together, these results suggest that the decrease in the annual incidence of *Salmonella*-infected broiler flocks in Sweden since 1991 is a result of the implementation of HACCP-based monitoring of feed, partly supported by the associated strengthened economic incentives for stringent implementation of monitoring in feed mills and broiler flocks and associated relevant interventions (Fig. 2).

Data from monitoring of high-risk feed ingredients before introduction to feed mills revealed that use by some mills of soymeal with undocumented *Salmonella* status resulted in a higher level of *Salmonella* contamination than at mills using *Salmonella*-safe soymeal (Fig. 3). *Salmonella* was transmitted via the contaminated soymeal into the feed production, as indicated by higher levels of *Salmonella* contamination in HACCP-based samples (Fig. 4). *Salmonella* was eventually also transmitted to the finished feed in the mills using nonsafe soymeal and subsequently infected 78 swine-producing herds (Wierup, 2006; Wierup and Häggblom, 2010). This finding is in line with experiences that vegetable proteins, typically soybean, are a high-risk feed ingredient for *Salmonella* contamination and that safe sourcing is an effective way to reduce that risk (EFSA, 2008; Wierup, 2016).

An additional measure applied in Sweden is that high-risk feed materials before entering feed mills must test negative for *Salmonella* contamination, and following treatment with organic acids for consignments tested positive (Koyuncu et al. 2013). The reason why the combined control procedures did not prevent contamination of the finished feed in Company A mills, in contrast to the mills of Companies B–E, was probably that the level of *Salmonella* contamination in the incoming feed ingredients was too high, in combination with suboptimal management of the HACCP monitoring procedure, indicated in a special inquiry (Wierup, 2006).

The results also show that the HACCP concept can be an effective tool to supply feed mills with *Salmonella*-safe feed ingredients. This was demonstrated for a Norwegian soybean crushing plant, which successfully produced *Salmonella*-safe soymeal over a 19-year period despite frequent (34%) and highly varied (92 different serovars) *Salmonella* contamination in samples from incoming soybean (Wierup and Kristoffersen, 2014). Similar results are also reported for a Swedish plant producing rapeseed meal (Jordbruksverket, 2021).

It should be emphasized that the achievements described above through use of the HACCP required interventions of relevant preventive biosecurity measures and corrective actions when the HACCP-based monitoring identified *Salmonella* contamination. This is in agreement with the EU guidance on implementation of HACCP (EC, 2005), which requires targets to be set for the overall reduction in prevalence of the zoonotic agent in question (here *Salmonella*) and critical limits at critical control points to distinguish acceptability from unacceptability, for successful prevention, elimination, or reduction of the identified hazard. Experiences from Sweden show that interventions are always necessary whenever *Salmonella* contamination is identified, as specified in the monitoring guidelines (Foder och Spannmål, 2022).

In addition, the use of economic incentives can be useful for reaching set targets. In Sweden, such incentives include a ban on delivering *Salmonella*-contaminated feed to farms and compulsory destruction of *Salmonella*-infected broilers and of *Salmonella*-contaminated food at the expense of the producer. As in broiler production, state compensation for those costs initially provided in the production of other livestock has gradually been reduced or fully removed (SVA, 2022). Nevertheless, the total costs for control of *Salmonella* in feed mills, including severe events requiring interventions and even rare stop of production, are estimated by the industry to represent <1% of the customer feed price (Wierup and Widell, 2014).

## Conclusion

The introduction in 1991 of a HACCP-based *Salmonella* control of feed production was followed by a considerable decrease in contaminated feed mill samples and in the annual incidence of *Salmonella* infected flocks to a level of <5 to 10, which has remained despite the production has increased from 39 to currently 112 million chickens. The previous endpoint testing failed to detect apparently low concentration of *Salmonella*, which readily can infect young chickens. The implementation of the HACCP concept also in crushing plants was found to supply feed mills with *Salmonella*-safe soy- and rapeseed meal as feed ingredients. The achievements from the use of the HACCP required interventions involving relevant preventive biosecurity measures and corrective actions when the HACCP-based monitoring identified *Salmonella* contamination.
